# The *Drosophila *cell adhesion molecule Neuroglian regulates Lissencephaly-1 localisation in circulating immunosurveillance cells

**DOI:** 10.1186/1471-2172-10-17

**Published:** 2009-03-25

**Authors:** Michael J Williams

**Affiliations:** 1 Institute of Biological and Environmental Sciences, University of Aberdeen, Tillydrone Avenue, Aberdeen AB24 2TZ, UK

## Abstract

**Background:**

When the parasitoid wasp *Leptopilina boulardi *lays its eggs in *Drosophila *larvae phagocytic cells called plasmatocytes and specialized cells known as lamellocytes encapsulate the egg. This requires these circulating immunosurveillance cells (haemocytes) to change from a non-adhesive to an adhesive state enabling them to bind to the invader. Interestingly, attachment of leukocytes, platelets, and insect haemocytes requires the same adhesion complexes as epithelial and neuronal cells.

**Results:**

Here evidence is presented showing that the *Drosophila *L1-type cell adhesion molecule *Neuroglian *(*Nrg*) is required for haemocytes to encapsulate *L. boulardi *wasp eggs. The amino acid sequence FIGQY containing a conserved phosphorylated tyrosine is found in the intracellular domain of all L1-type cell adhesion molecules. This conserved tyrosine is phosphorylated at the cell periphery of plasmatocytes and lamellocytes prior to parasitisation, but dephosphorylated after immune activation. Intriguingly, another pool of Nrg located near the nucleus of plasmatocytes remains phosphorylated after parasitisation. In mammalian neuronal cells phosphorylated neurofascin, another L1-type cell adhesion molecule interacts with a nucleokinesis complex containing the microtubule binding protein lissencephaly-1 (Lis1) [1]. Interestingly in plasmatocytes from *Nrg *mutants the nucleokinesis regulating protein Lissencephaly-1 (Lis1) fails to localise properly around the nucleus and is instead found diffuse throughout the cytoplasm and at unidentified perinuclear structures. After attaching to the wasp egg control plasmatocytes extend filopodia laterally from their cell periphery; as well as extending lateral filopodia plasmatocytes from *Nrg *mutants also extend many filopodia from their apical surface.

**Conclusion:**

The *Drosophila *cellular adhesion molecule Neuroglian is expressed in haemocytes and its activity is required for the encapsulation of *L. boularli *eggs. At the cell periphery of haemocytes Neuroglian may be involved in cell-cell interactions, while at the cell centre Neuroglian regulates the localisation of the nucleokinesis complex protein lissencephaly-1.

## Background

When the morphology of *Drosophila *haemocytes is compared, three types of cells can be identified: plasmatocytes, lamellocytes and crystal cells. Plasmatocytes resemble the mammalian monocyte/macrophage lineage and are involved in the phagocytosis or encapsulation of invading pathogens [2,3]. Lamellocytes are larger than the other haemocytes, are rarely seen in healthy larvae and seem to be specialized for the encapsulation of invading pathogens [4,5]. Crystal cells rupture to secrete components of the phenol oxidase cascade, involved in melanisation of invading organisms, wound repair and coagulation [6-8]

Endoparasitic wasps from the Hymenoptera family are known to parasitize *Drosophila *larvae. Once the invader is recognized as foreign circulating plasmatocytes somehow adhere and spread around the egg. After spreading the plasmatocytes form cellular junctions between the cells effectively separating the egg from the larval open circulatory system (hemoceol) [9,10]. Following plasmatocyte adherence and spreading, lamellocytes recognize the plasmatocytes surrounding the egg, and finally the capsule is melanised due to crystal cell rupture [9-11]. From these events it is obvious that adhesion and cell shape change are essential parts of the *Drosophila*'s cellular immune response against parasitoid wasp eggs.

Circulating immune surveillance cells need to remain mobile until they receive the correct signals to become adherent. In the case of *Drosophila *larvae, haemocytes change from non-adhesive circulating cells to adhesive non-circulating cells after parasitisation. Evidence is mounting that during attachment or encapsulation events leukocytes, platelets, and insect haemocytes use the same adhesion complexes as epithelial and neuronal cells [10,12-19]. In platelets the mammalian homolog of Neuroglian, L1-Cam is necessary for platelet-platelet interactions [20]. Furthermore in the tobacco hornworm *Manduca sexta *the L1-Cam family member Neuroglian has been shown to interact with integrins during immune encapsulation responses [17,18]. Because of these results I decided to look at the involvement of *Neuroglian *in the *Drosophila *cellular immune response against eggs from the parasitoid wasp *Leptopilina boulardi*.

## Results

### Neuroglian cellular localization

**Figure 1:**
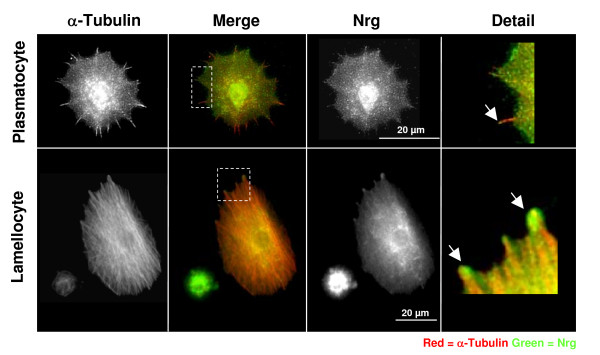
**Nrg expressed in plasmatocytes and lamellocytes**. Plasmatocytes and lamellocytes bled from control larvae 38–40 h post-parasitisation by L. boulardi G486 and co-stained with anti-Tubulin (red) and anti-Nrg (green). In the detail figure of plasmatocytes and lamellocyte arrows indicate that Nrg protein is enriched at the tips of some filopodia.

To begin to elucidate if *Drosophila Neuroglian (Nrg) *was involved in the cellular immune response haemocytes were bled from parasitized control larvae (*w*^1118^) approximately 40 hours after parasitisation and co-stained with anti-α-Tubulin and anti-Nrg antibodies [21]. In both plasmatocytes and lamellocytes bled from parasitized control larvae Nrg was expressed at the plasma membrane, and accumulated in filopodia at the cell periphery (Figure 1, arrows).

*Nrg *has two splice forms one of which, Nrg^180^, is specifically expressed in the nervous system [22]. To make sure that the Nrg protein expressed in haemocytes is not Nrg^180^, haemocytes bled from non-parasitized larvae, as well as from larvae 24 and 40 h post-parasitisation were stained with a mouse monoclonal antibody that specifically recognizes Nrg^180 ^[22]. No staining was observed in any of the haemocytes (data not shown), showing that the Nrg expressed in haemocytes is not Nrg^180^.

### Nrg-FIGQY dephosphorylated after parasitisation

**Figure 2:**
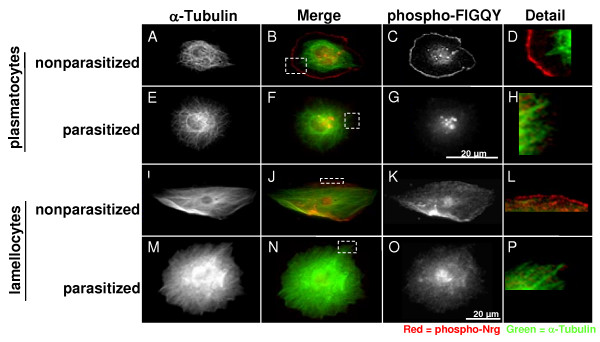
**Nrg-FIGQY phosphorylation regulated by immune activation (A-H) Plasmatocytes bled from non-parasitized control larvae or 38–40 h post-parasitisation, and stained with anti-α-Tubulin (A, E) and anti-phospho-FIGQY (C, G)**. (B) Merge of A and C, square denotes area shown in D. (D) Detail of the plasma membrane of a plasmatocyte from a non-parasitized larva. (F) Merge of E and G, square denotes area shown in H. (H) Detail of the plasma membrane of a plasmatocyte from a parasitized larva. (**I-P**) Lamellocytes bled from non-parasitized control larvae or 38–40 h post-parasitisation, and stained with anti-Tubulin (I, M) and anti-phospho-FIGQY (K, O). (J) Merge of I and K, square denotes area shown in L. (L) Detail of the plasma membrane of a lamellocyte from a non-parasitized larva. (N) Merge of M and O, square denotes area shown in P. (P) Detail of the plasma membrane of a plasmatocyte from parasitized larva.

Neuroglian belongs to the L1-family of cellular adhesion molecules, along with mammalian L1, Neurofascin, NRCAM, NgCAM and *Caenorhabditis elegans *LAD-1 [21,23,24]. All L1 family members have the conserved amino acid sequence FIGQY containing a tyrosine phosphorylation site in their intracellular domain. Dephosphorylation of the FIGQY tyrosine allows L1-type cellular adhesion molecules (CAMs) to interact with ankyrin, and through ankyrin to interact with the spectrin cortical-actin cytoskeleton [25]. Using an antibody raised against phospho-FIGQY [26] it was evident that in plasmatocytes bled from non-parasitized control larvae the conserved Nrg-FIGQY tyrosine was phosphorylated at the cell periphery (Figure 2B and 2D) and also at sites near the nucleus (Figure 2C). In plasmatocytes bled from larvae 40 h post-parasitisation there was virtually no phospho-tyrosine observed at the cell periphery (Figure 2E and 2H), while Nrg was still phosphorylated at sites near the cell centre (Figure 2G). In lamellocytes it was not as obvious that Nrg was phosphorylated at the plasma membrane in non-parasitized larvae (Figure 2J and 2L), though it was evident that there was less phosphorylation 40 h after parasitisation (Figure 2N and 2P). There was never any accumulated phospho-Nrg observed near the cell centre of lamellocytes bled from either non-parasitized or parasitized control larvae (Figure 2K and 2O).

### Neuroglian required for Lis1 perinuclear localization

**Figure 3:**
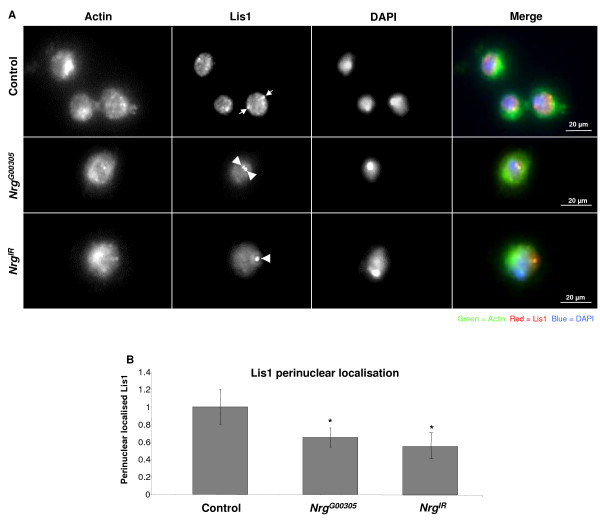
**Lis1 protein mislocalised in *Nrg *mutants**. (**A**) Haemocytes were bled from wandering third instar control (*w*^1118^), *Nrg*^*G*00305^, or *UAS*-*Nrg*^*IR*^*;He-Gal4 *larvae and stained for Lis1 expression (red) and actin (green), nuclei were visualised by DAPI staining (blue). In control cells Lis1 was observed tightly associated with the nucleus and at centriole-like structures also tightly associated with the nucleus (arrows). In plasmatocytes from *Nrg*^*G*00305 ^or *UAS*-*Nrg*^*IR*^*;He-Gal4 *larvae Lis1 was more diffuse throughout the cytoplasm, and in many cells Lis1 expression was enhanced at centriole-like structures (arrowheads). (**B**) Lis1 perinuclear expression levels. Haemocytes were bled from control, *Nrg*^*G*00305 ^or *UAS*-*Nrg*^*IR*^*;He-Gal4 *wandering third instar larvae. The haemocytes were stained for Lis1 expression. ImageJ was used to measure fluorescence intensity of Lis1 staining surrounding the nucleus of at least 75 haemocytes from three different larvae. Asterisks indicate a significant difference from control cells. (Student's *t*-test, *P *< 0.01).

**Figure 4:**
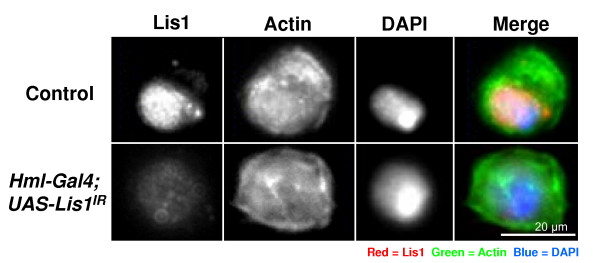
**Haemocytes were bled from non-parasitized control and *He-GA4/UAS-Lis1*^*IR *^larvae and stained for Lis1 (red) and Actin (green), nuclei were visualised by DAPI staining (blue)**. Size bar indicates 20 μm.

In rat neuroblastoma cells the phosphorylated FIGQY-domain of neurofascin is bound by doublecortin [1]. Doublecortin (Dcx) is a microtubule binding protein that when mutated causes a type of neuronal migration disorder known as X-linked lissencephaly [27]. Doublecortin has been shown to interact with another microtubule associated protein known as lissencephaly-1 (Lis1) [28], and together they are involved in regulating the movement of the nucleus of neuronal cells during migration [29]. Phosphorylation the phospho-FIGQY-Nrg near the nucleus of plasmatocytes could allow Nrg to interact with a Dcx-Lis1 complex. To test this possibility, plasmatocytes from non-parasitized control, *Nrg*^*G*00305 ^mutant larvae, or larvae overexpressing an *Nrg *RNAi construct (*UAS-NrgIR*, from now on referred to as *Nrg*^*IR*^) specifically in haemocytes using the haemocyte-specific driver *Hemese-Gal4 *(*He-Gal4*) [30], were stained for Lis1 expression using an antibody raised against Human Lis1. Null mutations of *Nrg *are homozygous lethal, so the *Nrg*^*G*00305 ^allele which survives to adulthood was chosen for this study [31]. *Nrg*^*G*00305 ^may be a weak hypomorph with enough function to survive embryogenesis [32]. In plasmatocytes bled from control larvae Lis1 was observed surrounding the nucleus and at what could be the centrioles (Figure 3A, arrows, and 3B). In *Nrg*^*G*00305 ^or *Nrg*^*IR*^*;He-Gal4 *plasmatocytes less Lis1 was observed surrounding the nucleus, and its expression looked more diffuse throughout the cytoplasm than in controls (see Figure 3A, merged image). In these same cells Lis1 protein was enriched at what appeared to be perinuclear centriole-like structures that were not observed in control plasmatocytes (Figure 3A, arrowheads). To make sure that the antibody was specifically recognizing *Drosophila *Lis1 we crossed *He-Gal4 *to flies overexpressing a Lis1 RNAi construct (*UAS-Lis1*^*IR*^). Lis1 expression was significantly reduced in *He-Gal4/UAS-Lis1*^*IR *^haemocytes (Figure 4).

**Figure 5:**
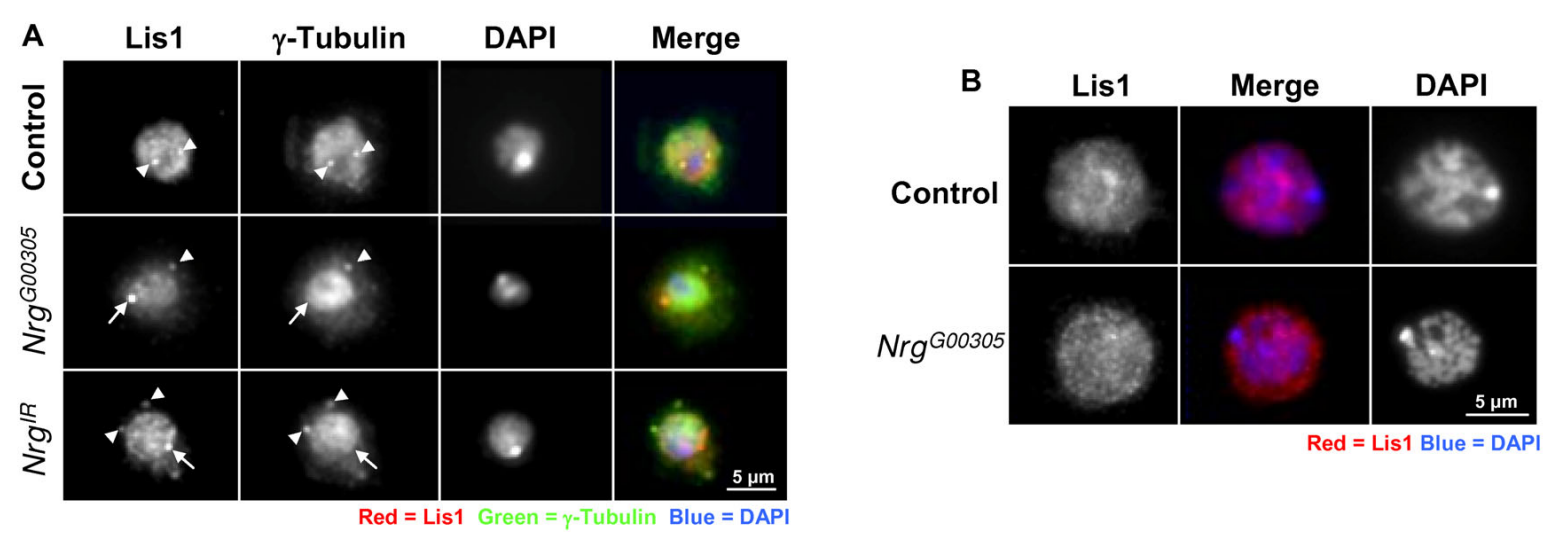
**(A) Haemocytes were bled from non-parasitized control larvae and stained for Lis1 (red) and γ-Tubulin (green), nuclei were visualised by DAPI staining (blue)**. Arrowheads indicate co-localisation of Lis1 and γ-Tubulin at the centrioles. Open-arrows indicate Lis1 specific expression seen in *Nrg*^*G*00305 ^and *UAS*-*Nrg*^*IR*^*;He-Gal4 *plasmatocytes. (**B**) Lamellocytes from parasitized control, and *Nrg*^*G*00305 ^larvae stained for Lis (red) and the nuclei were visualised by DAPI staining (blue).

To test if the Lis1-localised structures observed in *Nrg*^*G*00305 ^and *Nrg*^*IR*^*;He-Gal4 *plasmatocytes were centrioles plasmatocytes were co-stained with anti-Lis1 and anti-γ-tubulin. In control plasmatocytes Lis1 and γ-tubulin co-localised at the centrioles (Figure 5A, arrowheads). In plasmatocytes recovered from *Nrg*^*G*00305 ^or *Nrg*^*IR*^*;He-Gal4 *mutant larvae Lis1 and γ-tubulin still co-localised at the centrioles (Figure 5A, arrowheads). Interestingly, γ-tubulin did not localise to the Lis1 enriched structure observed in *Nrg*^*G*00305 ^or *Nrg*^*IR*^*;He-Gal4 *plasmatocytes (Figure 5A, arrows). In lamellocytes bled from parasitized control, *Nrg*^*G*00305 ^or *Nrg*^*IR*^*;He-Gal4 *larvae Lis1 expression was localised around the nucleus (Figure 5B). Interestingly, in lamellocytes from *Nrg*^*G*00305 ^or *Nrg*^*IR*^*;He-Gal4 *larvae Lis1 protein was still tightly localised around the nucleus (Figure 5B, and data not shown). This result may not be surprising as lamellocytes contain polytene chromosomes, are fully differentiated, and no centrioles were observed (data not shown).

### In *Nrg*^*G*00305 ^Nrg still present at plasma membrane

**Figure 6:**
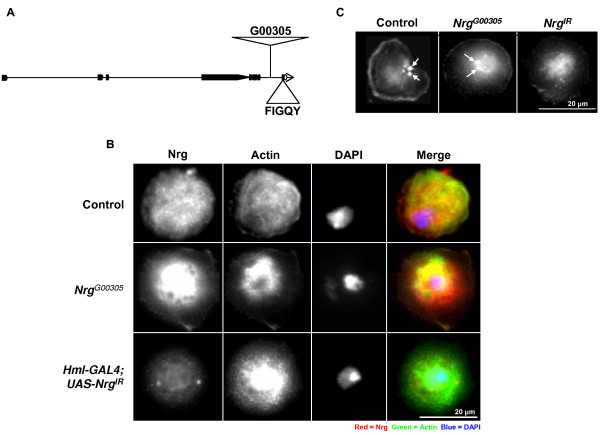
**FIGQY phosphorylation in *Nrg*^*G*00305 ^mutants**. (**A**) Schematic drawing of the *Nrg *gene indicating where the G00305 P-element is inserted. (**B**) Haemocytes were bled from non-parasitized control *Nrg*^*G*00305 ^and *UAS*-*Nrg*^*IR*^*;He-Gal4 *wandering third instar larvae and stained for Nrg expression. Nrg (red), Actin (green), nuclei were visualised by DAPI staining (blue). (**C**) Haemocytes were bled from non-parasitized control *Nrg*^*G*00305 ^and *UAS*-*Nrg*^*IR*^*;He-Gal4 *wandering third instar larvae and stained for phospho-FIGQY-Nrg. Arrows indicate phosphorylated Nrg near the nucleus.

The P-element insert that created the *Nrg*^*G*00305 ^allele inserted into the intron prior to the exon containing the FIGQY amino acid sequence (Figure 6A) [31]. The design of the P{PTT-GA} P-element allows for the incorporation of green fluorescent protein (GFP) into the open reading frame of Nrg, creating a chimeric protein [31]. To see if the insertion of GFP in the intracellular domain affected Nrg expression, Control and *Nrg*^*G*00305 ^haemocytes were collected form non-parasitized third instar larvae and stained for Nrg expression. No difference in Nrg expression or localisation was observed when *Nrg*^*G*00305 ^haemocytes were compared to controls (Figure 6B). Furthermore, *UAS-Nrg*^*IR*^*;He-Gal4 *haemocytes Nrg expression was significantly reduced compared to control cells (Figure 6B). Next, to test if the insertion of GFP in the intracellular domain affected Nrg-FIGQY phosphorylation, haemocytes from non-parasitized control and *Nrg*^*G*00305 ^mutants were stained with anti-phospho-FIGQY antibodies. In control and *Nrg*^*G*00305 ^mutant plasmatocytes phosho-FIGQY-Nrg was present at the cell periphery (Figure 6C) and near the nucleus (Figure 6C, arrows). Plasmatocytes bled from *UAS-Nrg*^*IR*^*;He-Gal4 *larvae had less phosho-FIGQY-Nrg at the cell periphery, and very little phosho-FIGQY-Nrg was observe near the cell centre. From this result I conclude that the GFP insert in the intracellular domain does not affect Nrg localisation or the phosphorylation of the FIGQY conserved tyrosine.

### Neuroglian needed for encapsulation

**Figure 7:**
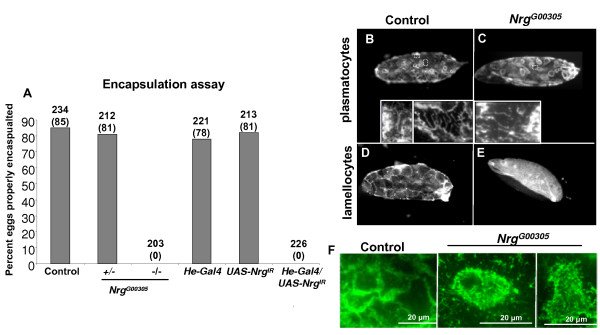
***Nrg *necessary for proper encapsulation of *L. boulardi *eggs (A) Encapsulation capacities of *Nrg *mutant larvae in response to parasitisation by *L. boulardi *G486**. [(Number of larvae with properly melanized wasp eggs/number of parasitized larvae) × 100)]. Numbers above the bars indicate the number of wasp parasitized larvae examined, numbers in parentheses indicate percentage of larvae that properly encapsulated the *L. boulardi *G486 eggs (**B, C**) Encapsulated wasp eggs recovered from larvae 22–24 h after parasitisation, and stained with the plasmatocyte specific protein Nimrod. (B) Control (*w*^1118^) and (C) *Nrg*^*G*00305 ^homozygous mutants. (**D, E**) Haemocytes 38–40 h post-parasitisation stained with the L1 lamellocyte specific antibody. (D) Control and (E) *Nrg*^*G*00305 ^homozygous mutants. (**F**) Control and *Nrg*^*G*00305^plasmatocytes attached to L. boulardi G486 eggs. Unlike controls, plasmatocytes from *Nrg*^*G*00305 ^extend filopodia from their apical side.

An encapsulation assay was performed on larvae parasitized by the avirulent *L. boulardi *wasp strain G486. When the avirulent wasp strain G486 parasitizes larvae a darkened cellular capsule is visible in the hemoceol 30–40 h later. While 81% of *Nrg*^*G*00305 ^heterozygous mutant larvae properly encapsulated and melanised *L. boulardi *eggs, *Nrg*^*G*00305 ^homozygous larvae never properly encapsulated the wasp egg (Figure 7A). Similar to *Nrg*^*G*00305 ^homozygous mutants, none of the larvae expressing *Nrg *RNAi in haemocytes properly encapsulated the wasp egg (Figure 7A).

In the encapsulation assay a larva is considered to have a defective cellular immune response when the wasp egg is not melanised. Melanisation is the final event in encapsulation, so this assay is not able to define the actual defect during the encapsulation process. To gain a better understanding of when the activity of Nrg is required during encapsulation, wasp eggs were recovered from either control or homozygous *Nrg*^*G*00305 ^mutant larvae at various times after parasitisation and stained for haemocyte specific markers. In general plasmatocytes attach to the wasp egg between 6–24 h after the egg is laid in the larval hemoceol [11]. To look at plasmatocytes during the encapsulation process wasp eggs were dissected from larvae 22–24 h post-parasitisation and stained for the plasmatocyte specific protein Nimrod [33,34]. By 22–24 h post-parasitisation wasp eggs recovered from control larvae (*w*^1118 ^or *Nrg*^*G*00305^/+) were completely encapsulated by plasmatocytes that had spread around the chorion (Figure 7B). Plasmatocytes that had not made cell-cell contacts sent out filopodia from the cell periphery towards other plasmatocytes (Figure 7B, inset). Eggs recovered from homozygous *Nrg*^*G*00305 ^mutants also had plasmatocytes attached to the wasp egg that had spread on the chorion. In most instances fewer plasmatocytes were attached to the egg than in control larvae and in some cases almost no plasmatocytes were attached (Figure 7C and data not shown). Nrg mutant plasmatocytes attached to the wasp egg also extended filopodia laterally from their cell periphery (Figure 7C, inset); yet unlike controls, *Nrg*^*G*00305 ^mutant plasmatocytes projected many filopodia from their apical side, giving the cells a fuzzy appearance (Figure 7F).

After plasmatocytes spread around the wasp eggs, lamellocytes recognize and attach to the plasmatocytes between 24–40 h after the wasp egg is laid in the hemoceol [9-11]. To study lamellocytes, wasp eggs were recovered from larvae approximately 38–40 h post-parasitisation and stained with the lamellocyte specific antibody L1 [33]. By 38–40 h post-parasitisation eggs recovered from control larvae were completely surrounded by fully spread lamellocytes (Figure 7D). In most *Nrg*^*G*00305 ^homozygous mutant larvae no lamellocytes were attached to wasp eggs, but in a few larvae a couple of lamellocytes were attached to the egg (Figure 7E, and data not shown).

## Discussion

The *Drosophila *cellular adhesion molecule Neuroglian is expressed in haemocytes and its activity is required for them to properly encapsulate eggs from the parasitiod wasp *L. boulardi*. It is possible that Nrg plays multiple roles when plasmatocytes adhere and spread on wasp eggs. At the cell periphery it could be involved in cell-cell interactions, while at the cell centre Nrg may regulate the localisation of a lissencephaly-1 containing complex.

In Drosophila larvae reduced Neuroglian activity caused an impairment of haemocyte adhesion to the wasp egg and reduced cell-cell interactions between plasmatocytes and lamellocytes. Similar to encapsulation events in *M. sexta *these two steps of encapsulation may require heterophilic interaction between Nrg and integrins, Nrg homophilic binding, or both [17,18]. In addition, both events may be accompanied by dephosphorylation of FIGQY to allow interaction of Nrg with ankyrin. Nrg may become dephosphorylated at the cell periphery to allow it to interact with Ankyrin protein and thus to the spectrin cortical-cytoskeleton [25,26]. Nrg has been localised to septate junctions in *Drosophila *embryonic epithelial cells and its activity is necessary for septate junction formation [35]. In the cellular immune response against parasitoid wasp eggs Nrg may be necessary for plasmatocytes to form cellular junctions during the encapsulation response. Once plasmatocytes spread around the wasp egg and make cell-cell contacts they form cellular junctions [9]. These junctions have been described as looking like septate junctions, and at least one septate junction protein, Coracle, has been localised to the cell-cell interactions of plasmatocytes on wasp eggs [10].

**Figure 8:**
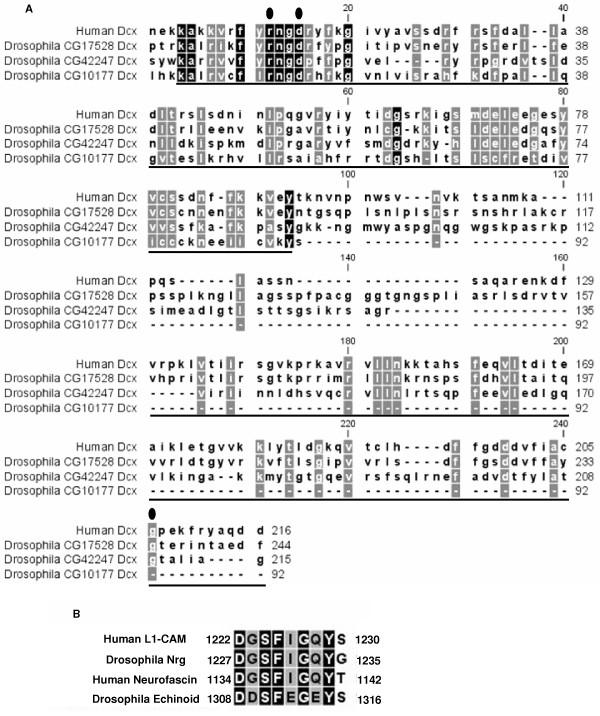
**Sequence alignments (A) Sequence alignment of three *Drosophila *proteins containing Dcx-domains and human doublecortin**. Underlined sequences indicated Dcx domains, filled circles indicate the three amino acids shown to be necessary for doublecortin to interact with neurofascin. (**B**) Sequence alignment comparing the FIGQY domains of Human L1-CAM, Human Neurofascin and *Drosophila *Neuroglian with *Drosophila *Echinoid.

In neurons it has been shown that the L1-family member neurofascin interacts with doublecortin and this interaction is necessary for neuronal migration [1]. Doublecortin is a microtubule-associated protein involved in neuronal migration [29], and along with the microtubule array and neurofascin, doublecortin interacts with lissencephaly-1 (LIS1). In both mammalian and *Drosophila *neurogenesis, Lis-1 is necessary for neuroblast proliferation and migration [36-38]. The doublecortin-Lis1 interaction is necessary for nucleokinesis during neuronal migration [39]. It is speculated that the interaction with neurofascin may be necessary to anchor the Lis1 complex to generate the force necessary for nucleokinesis, and without the signal from neurofascin nucleokinesis and cell migration cannot occur [1,36]. It may be that phosphorylation of the Nrg-FIGQY tyrosine at the plasmatocyte cell centre is necessary for Nrg to interact with a *Drosophila *doublecortin domain (Dcx) containing protein, to allow for nuclear anchoring. In an *Nrg*^*G*00305 ^mutant plasmatocyte Nrg may not be able to interact with a Dcx-domain protein, thus the Dcx-Lis1 complex cannot interact properly with the nuclear membrane and ends up at the unidentified perinuclear centriole-like structures or diffuse in the cytoplasm. There is no obvious homolog of mammalian doublecortin in *Drosophila*, but there are three proteins that contain Dcx domains, two of which are very similar to other doublecortin domain proteins called doublecortin-like kinase-1 and -2 in mammals. In a study to define the interaction of doublecortin with neurofascin, three amino acids in doublecortin were discovered to be important for this interaction [1]. Interestingly, all three of these amino acids are conserved in the *Drosophila *doublecortin-like kinase homolog CG17528 and the Dcx-domain protein CG42247, while two are conserved in the doublecortin-like kinase homolog CG10177 (Figure 8A). The possibility that Nrg interacts with one of these Dcx-domain containing proteins to anchor the nucleus during plasmatocyte spreading is currently under investigation. Of further interest is the observation that CG42247 was found to interact with another cell adhesion molecule Echinoid in a yeast-two hybrid screen [40]. Though Echinoid does not contain the FIGQY sequence found in L1-family molecules it does contain a similar sequence, FEGEY, in its intracellular domain near the C-terminus (Figure 8B).

**Figure 9:**
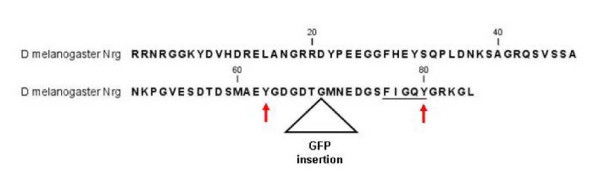
**Neuroglian^167 ^intracellular domain showing the conserved FIGQY sequence (underline) and the two predicted tyrosine phosphorylation sites (arrows)**. The location of the inserted GFP sequence is also indicated. Phosphorylation of the conserved tyrosines was predicted using the Netphos 2.0 program.

In *Nrg*^*G*00305 ^mutants the FIGQY tyrosine is still phosphorylated yet Lis1 mislocalises in the mutant plasmatocytes. This leads to the possibility that phosphorylation of the FIGQY tyrosine is not sufficient for interaction of Nrg with the Lis1 complex. In the study where neurofascin was shown to interact with doublecortin, it was shown that doublecortin only slightly bound to L1-CAM, and not at all to NRCAM, even though they also contain phospho-FIGQY [1]. There is another conserved tyrosine found in all L1-family members upstream of the FIGQY sequence (Figure 9). This tyrosine is predicted to be phosphorylated in both Neuroglian and neurofascin, but not in L1-CAM or NRCAM. The GFP sequence the *Nrg*^*G*00305 ^allele is incorporated between these two conserved tyrosines and may disrupt their interaction with the Lis1 complex. Also, even though it was shown that phospho-FIGQY was necessary for the interaction of neurofascin with doublecortin *in vivo*, it may not be sufficient [1]. Another possibility is insertion of GFP into the Nrg open reading frame may change the conformation of the intracellular domain blocking the interaction of Nrg with the Lis1 complex even though the conserved FIGQY tyrosine is phosphorylated.

## Conclusion

Most of what we understand about the complex that regulates nucleokinesis comes from studies on neuronal development [29]. Yet, one of the first proteins discovered to regulate nucleokinesis, lissencephely-1/NUDF was discovered in T cells [41]. Leukocytes migrating through interstitial tissues must solve many of the same problems as neurons migrating during development, one of the main problems being nuclear migration. What is not fully understood in neurons, and not studied at all in the cellular immune response, is exactly how the nucleokinesis complex regulates nuclear migration. Here evidence was presented showing that in one subtype of *Drosophila *circulating immunosurveillance cells a transmembrane molecule, Neuroglian, somehow regulates the localisation of at least one nucleokinesis complex protein, Lis-1. What has not been elucidated is the significance of this in nucleokinesis or if this aspect of Neuroglian function is important for cellular immune response function. Still, it is intriguing to speculate that similar to neuronal cells, immunosurveillance cells also use the nucleokinesis apparatus to regulate nuclear migration.

## Methods

### Insects

*Drosophila *strains *w*^1118 ^and *Nrg*^*G*00305 ^were obtained from the Bloomington Stock Centre. *Hemese-GAL4 *driver line has been described previously [30]. *UAS-Nrg*^*IR *^strain number 27201 and *UAS-Lis1*^*IR *^strain number 6216 were obtained from the Vienna *Drosophila *RNAi Centre (VDRC). Flies were kept on a standard corn molasses meal diet at between 21–25°C. The G486 strain of *L. boulardi *was bred on a *w*^1118 ^stock of *D. melanogaster *at room temperature using a standard medium. Adult wasps were maintained at room temperature on grape juice agar.

### Wasp egg encapsulation assay

The encapsulation assay was done according to Sorrentino et al., [5]. Briefly, two days before parasitisation the appropriate fly strains were crossed and kept at 21–25°C. Four or five females of *L. boulardi *G486 were allowed to infest at room temperature for 2 hours, after which the *Drosophila *larvae were transferred to apple juice plates and left at room temperature for 40–42 hours. After this time the larvae were collected, washed in PBS, and then viewed under a stereomicroscope for the presence of a dark capsule. Larvae in which no dark capsule was observed were dissected in 20 μl of PBS to determine if they had been parasitized. Larvae containing eggs from the parasitoid that hadn't darkened by this time were scored as non-encapsulated. Non-parasitized larvae were excluded from the count.

### Antibodies and reagents

Lamellocyte specific mouse monoclonal antibody (L1a) [33] and plasmatocyte specific monoclonal mouse anti-Nimrod [33,34] were used undiluted, mouse monoclonal antibody anti-α-Tubulin (Sigma) was diluted 1:1,000, rabbit polyclonal anti-α-Tubulin (Abcam) was diluted 1:500, mouse monoclonal anti-γ-Tubulin (Sigma) was diluted 1:500, rabbit polyclonal anti-phospho-FIGQY was diluted 1:250 [26], mouse monoclonal anti-Nrg 3C1 was diluted 1:1,000 [21,23], rabbit polyclonal anti-Lis1 (Abcam, ab2607) was diluted 1:500, and mouse monoclonal anti-Nrg^180 ^(BP104, Developmental Studies Hybridoma Bank) was used undiluted.

### Immunofluorescence

#### Wasp egg staining

For lamellocyte monoclonal antibody (L1a) and the plasmatocyte specific monoclonal antibody (P1a), wasp eggs were bled from larvae, into 20 μl of phosphate buffered saline (PBS), and allowed to attach to a glass slide (SM-011, Hendley-Essex, Essex, UK) for 5 minutes at room temperature. Staining and analysis were done according Williams et al., [10].

#### Circulating haemocyte staining

For all haemocyte antibody staining, haemocytes were bled from a larvae into 20 μl of PBS, and allowed to attach to a glass slide (SM-011, Hendley-Essex, Essex, UK) for 1 hour. Staining and analysis were done according to Williams et al., [42]. Cells were visualized using a Zeiss Axiovert 200 M epifluorescent microscope and digital pictures were taken with a Hamamatsu C4742-80-12AG video unit, controlled by the Simple PCI 6.1 program (Hamamatsu). ImageJ (NIH) was used for digital editing. ImageJ was used to measure fluorescent intensity.
